# Hedgehog-related gene *wrt-10* mediates intergenerational effects of fasting on larval size in *Caenorhabditis elegans*

**DOI:** 10.17912/micropub.biology.002269

**Published:** 2026-08-06

**Authors:** Saige K. Motkoski, Emma Rossander, Maxwell Schweigel, Muskan Karmani, Nicole M. Templeman

**Affiliations:** 1 Biology, University of Victoria, Victoria, BC, Canada

## Abstract

Low food availability can cause delays in reproductive aging to allow for eventual improvements in conditions, or might promote investment in offspring to increase their survival. Here, we tested whether *Caenorhabditis elegans* Hedgehog-related *wrt-10* mediates these responses. We found that reproductively aged wild-type worms and *wrt-10 ( aus36 )* null mutants both had a greater likelihood of progeny production after 24-h or 48-h fasting periods in early adulthood, indicating that *wrt-10* is not required for this response. However, unlike wild-type, *wrt-10 ( aus36 )* mutants did not produce longer larvae after fasting. Therefore, *wrt-10* may enable diet-restricted *C. elegans* adults to channel resources towards their progeny.

**Figure 1. Effects of early-adulthood fasting on late-mated reproductive capacities and larval offspring body lengths, in wild-type N2 worms and wrt-10(aus36) null mutants:** ( **A** ) Experimental design showing timing of 24-h and 48-h fasting periods and subsequent assays. Hypochlorite-synchronized wild-type N2 worms and *wrt-10 ( aus36 )* mutants were raised on free-feeding conditions until day 2 of adulthood, when they were distributed between OP50-seeded plates (free-feeding groups) or unseeded plates (fasting groups); after 24-h or 48-h without food, fasted worms were returned to OP50-seeded plates. For late-mating assays, each hermaphrodite was individually plated at day 6 of adulthood with three *fog-2 ( q71 )* males for 72 h. For measuring larval offspring lengths, hermaphrodites were instead bleached at day 4 of adulthood, after 48-h of fasting or free-feeding; collected eggs were raised on OP50-seeded plates for 14 h, and then L1 larval lengths were measured. ( **B, C** ) Exposure to 24-h and 48-h fasting periods (starting at day 2 of adulthood) increases the proportion of the population capable of producing viable progeny after mating at day 6 of adulthood, for wild-type N2 worms ( **B** ) and *wrt-10 ( aus36 )* mutants ( **C** ). 4-5 independent populations per strain (signified by scatter points and line connectors) were distributed across experimental groups, with 13-103 worms per group for each replicate population after censoring. ( **D, E** ) For wild-type N2 worms, 48 h of early-adulthood fasting increases the mean body length of the L1 larval offspring ( **D** ). The mean difference (Δ) in L1 larval length for each population in response to the parental 48-h fasting period is also presented ( **E** ). 4 independent populations (signified by circle scatter points) were distributed across groups, and average L1 length was calculated for 14-35 larvae per group for each population. Triangle scatter points indicate the mean of the difference between the average offspring larval length of the free-fed and the 48-h-fasted groups (each triangle scatter point indicates an independent population). ( **F, G** ) For *wrt-10 ( aus36 )* mutants, 48 h of early-adulthood fasting does not have a statistically significant effect on the mean body length of the L1 larval offspring ( **F** ). The mean difference (Δ) in L1 larval length for each population in response to the parental 48-h fasting period is also presented ( **G** ). 4 independent populations (signified by circle scatter points) were distributed across groups, and average L1 length was calculated for 10-35 larvae per group for each population. Triangle scatter points indicate the mean of the difference between the average offspring larval length of the free-fed and the 48-h-fasted groups (each triangle scatter point indicates an independent population). ∗ p ≤ 0.05, ∗∗ p < 0.01, ∗∗∗ p < 0.001, ∗∗∗∗ p < 0.0001 compared to free-fed groups. Error bars represent SEM.

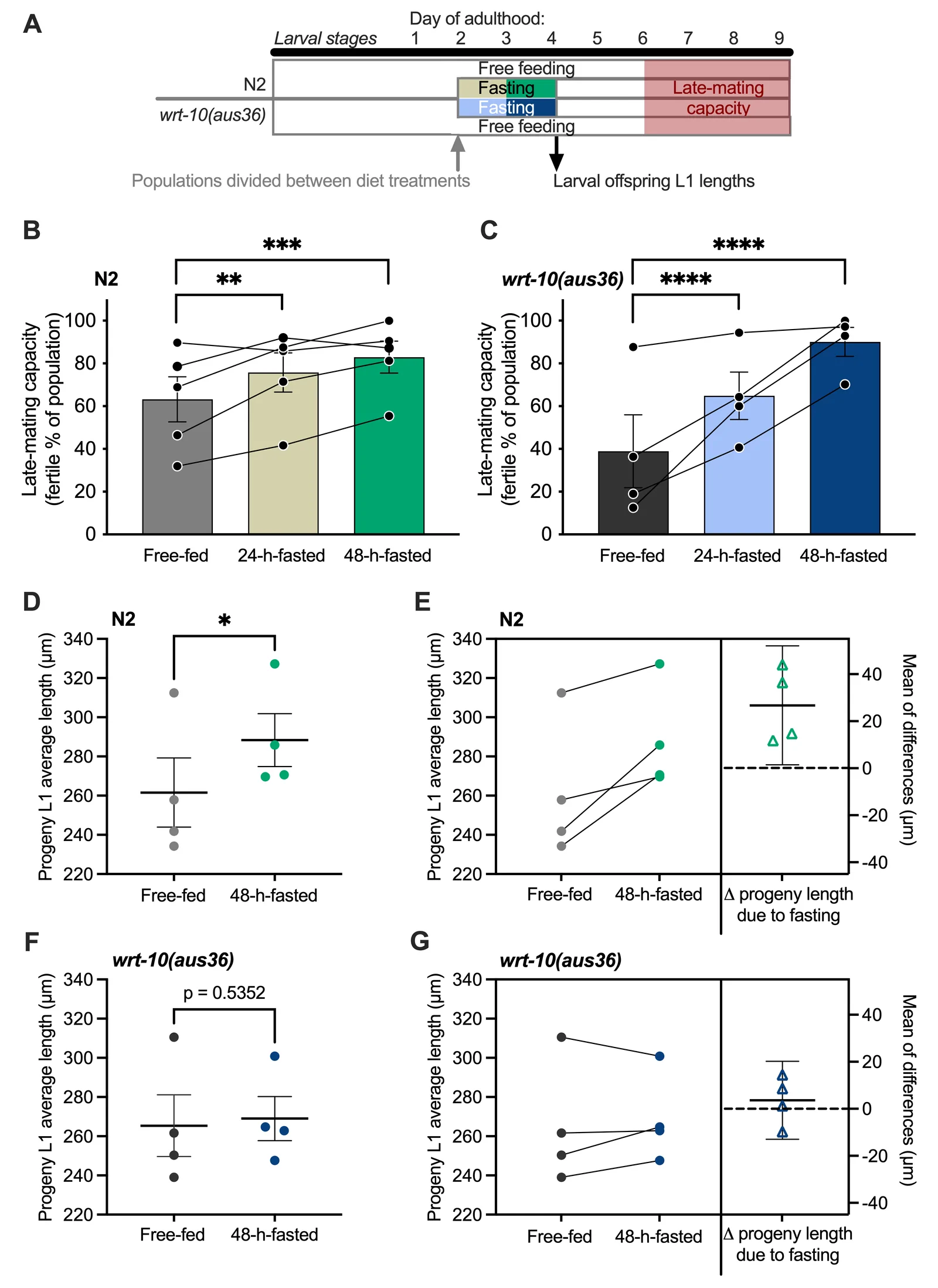



## Description

Eating patterns and nutrient conditions have far-reaching consequences that can include changes to lifespan, reproductive longevity, and progeny traits. When faced with environmental stressors like limited food, organisms can undergo physiological shifts to increase somatic tissue maintenance and suppress immediate progeny production, allowing them to delay reproduction until favorable conditions are encountered (Williams 1966; Crawford et al. 2007; Duffield et al. 2017). *C. elegans* demonstrate this life history strategy in a drastic fashion with the non-reproductive larval arrest, dauer diapause, or adult reproductive diapause phenotypes that arise when food is restricted during development (Cassada and Russell 1975; Johnson et al. 1984; Angelo and Gilst 2009). Also in line with this strategy, an intermittent fasting regime in early adulthood decreases the number of progeny from wild-type *C. elegans* hermaphrodites during the dietary intervention period, but subsequently leads to more progeny produced by hermaphrodites mated late in their reproductive window (Sultanova et al. 2025). Similarly, *eat-2 ( ad465 )* or *eat-2 ( ad1116 )* models of dietary restriction produce fewer progeny early in adulthood, but significantly extend their reproductive span (Crawford et al. 2007; Hughes et al. 2007).

Alternatively, shunting resources towards reproductive outputs at some cost to the parental soma may confer a fitness benefit under diet-restricted conditions, by increasing the likelihood that offspring are sufficiently provisioned to survive the unfavorable conditions or seek new food sources (Clutton-Brock 1984; Duffield et al. 2017). Adult *C. elegans* exposed to nutrient depletion also utilize elements of this strategy. For instance, restricting bacterial food concentrations for the parental generation led to an increase in average egg size, and the progeny of the diet-restricted hermaphrodites were less likely to develop into dauer larvae (Harvey and Orbidans 2011). Similarly, Hibshman et al. (2016) found that diet-restricted *C. elegans* hermaphrodites produced fewer but larger larvae that were resistant to the detrimental impacts of L1 larval starvation on fecundity, and observed that *eat-2 ( ad465 )* embryos were longer than wild-type embryos.

Here, our goal was to test the role of the *C. elegans* hedgehog-related signaling factor WRT-10 in mediating strategic responses to dietary restriction, since *wrt-10* has been implicated in both postponing reproductive aging and increasing the maternal costs of reproduction. Of note, starvation causes Drosophila larvae to elevate Hedgehog (Hh) production, leading to a promotion of their survival by regulating a range of nutrient availability responses, including lipid mobilization, growth, and developmental timing (Rodenfels et al. 2014). Adult *C. elegans* hermaphrodites also show a whole-body upregulation of hedgehog-related *wrt-10* expression after 9 h of fasting, an upregulation that is maintained up to at least 48 h of fasting (Uno et al. 2013). In free-feeding *C. elegans* , hypodermal overexpression of *wrt-10* significantly delays age-related reproductive decline (Templeman et al. 2020). Additionally, *wrt-10* upregulation in the germline promotes mating-induced shrinking and premature death of *C. elegans* hermaphrodites, phenotypes that may represent detrimental somatic consequences of channeling resources towards reproduction (Shi and Murphy 2023). Here, we hypothesized that *wrt-10* might contribute towards the delay in maternal reproductive decline (Sultanova et al. 2025) and/or the increase in progeny size (Hibshman et al. 2016) that can be induced when *C. elegans* adults are exposed to a period of dietary restriction.

Using wild-type N2 *C. elegans* and *wrt-10 ( aus36 )* mutants with a premature stop codon in the first *wrt-10* exon (Sherry et al. 2019), we evaluated how removing food from day 2 adults for 24 h or 48 h (before a return to free-feeding conditions) affected the capacity of reproductively aged day 6 hermaphrodites to produce viable progeny when mated to young males ( Figure 1A ). For our dietary restriction manipulation, we used a repeated washing technique to remove live bacteria from adult *C. elegans* before placing them on plates without their bacterial food source for the fasting periods (Walker et al. 2021). Both wild-type and *wrt-10 ( aus36 )* hermaphrodites had a significantly greater likelihood of late-mating success if they had experienced a 24-h or 48-h fasting period in early adulthood ( Figure 1B, 1C), pointing to a delay in reproductive aging caused by the dietary restriction manipulation. Thus, *wrt-10 ( aus36 )* mutants appeared capable of postponing their reproductive decline after experiencing dietary restriction, despite their putative null mutation of *wrt-10* (Sherry et al. 2019).

Next, we tested how 48 h of fasting, from day 2 to day 4 of adulthood, affected the average body lengths of the L1 larvae produced immediately after the parental fasting period ( Figure 1A ). After a 48-h fasting period, wild-type hermaphrodites produced eggs that developed into longer L1 larvae on average, compared to the subsets of the wild-type populations that were continuously free-fed ( Figure 1D, 1E). This fasting-induced response (consistent with previous reports in wild-type worms; (Hibshman et al. 2016)) was not seen in *wrt-10 ( aus36 )* mutants, whose L1 larvae had comparable lengths regardless of whether their parent had been free-fed or fasted for 48-h ( Figure 1F, 1G). Functional *wrt-10* expression may therefore help mediate the larval size response to parental dietary restriction.

In summary, two alternative life history strategies are thought to increase fitness when an organism is faced with low food availability: 1) preserving capabilities for reproduction at a later point when conditions are favorable; or 2) channeling resources into reproduction at some cost to the soma, to increase the probability of offspring survival and success (Williams 1966; Clutton-Brock 1984; Duffield et al. 2017). Since we found that populations of *wrt-10 ( aus36 )* mutants were highly adept at improving their late-mating capacity in response to early-adulthood fasting, functional *wrt-10* expression is not required for the delay in reproductive decline that is induced by dietary restriction in adulthood. On the other hand, *wrt-10 ( aus36 )* mutants did not respond to a 48-h fasting period by producing significantly larger progeny, unlike wild-type N2 *C. elegans* . Therefore, WRT-10 may be a relevant signaling factor for enabling diet-restricted *C. elegans* adults to channel resources towards their progeny. This aligns with its role in promoting the post-mating shrinking and early death of mated hermaphrodites, as these costs of *C. elegans* germline hyperactivity are also associated with provisioning progeny with adequate nutritional resources (Shi and Murphy 2023). These results suggest that the hedgehog-related *wrt-10* signaling pathway in adult *C. elegans* could be involved in stimulating greater maternal investment in progeny under conditions of dietary restriction.

## Methods

***C. elegans* maintenance:**

Strains were obtained from the Caenorhabditis Genetics Center (CGC) and cultured using standard methods (Brenner 1974). The N2 Bristol strain was used as wild-type, the *wrt-10 ( aus36 )* strain with a deletion predicted to cause a premature stop codon in the first *wrt-10* exon were used as *wrt-10* putative null mutants (Sherry et al. 2019), and *fog-2 ( q71 ) V* males were used for late-mating experiments. *C. elegans* were cultured at 20 °C on Nematode Growth Media (NGM; Brenner 1974) for experimental assays, with *Escherichia coli* OP50 as the food source. Hypochlorite synchronization was performed by exposing gravid hermaphrodites to bleach solution (80 mL ddH2O, 5 mL 5M KOH, 15 mL sodium hypochlorite) followed by repeated rinsing in M9 buffer (Brenner 1974) before depositing eggs on OP50-seeded NGM plates, for age synchronization of experimental populations.

Replicates of all experiments were performed in a minimum of four independent populations, raised from separate starting populations.

**Fasting:**

On day two of adulthood, all hermaphrodites were washed five times in M9 buffer before assignment to fasting or free-fed experimental groups. Specifically, they were washed off plates with M9 buffer and left to settle at the bottom of an upright 15 mL conical centrifuge tube for 2-5 minutes before aspiration of supernatant and resuspension of worms in M9 buffer; this process was repeated for a total of five washes to prevent carry-over of live bacteria onto fasting plates (Walker et al. 2021). After the final wash, some individuals were placed on OP50-seeded NGM plates as the free-feeding group, whereas worms assigned to fasting groups were placed on unseeded, room-temperature NGM plates. After 24 h, all groups were transferred: control groups were maintained on plates seeded with OP50, the 24-h fasting groups were returned to plates seeded with OP50, and the 48-h fasting groups were transferred to fresh unseeded NGM plates. After another 24 h, all groups were transferred to seeded plates for subsequent late-mating assays, or bleached to measure offspring larval size.

**Late-mating assays:**

On day 6 of adulthood, hermaphrodites were separated onto individual 35 mm plates at a 1:3 ratio with day 1 adult *fog-2 ( q71 ) V* males for 72 h. Hermaphrodites that had progeny on the plate after this period were scored as fertile ( *i.e.* , reproductively capable); plates with dead or missing hermaphrodites, matricidal bagging, absent males, or contamination were censored.

**Larval size:**

On day 4 of adulthood, immediately following the 48-h fasting period, hermaphrodites were washed off the plates with M9 buffer and lysed with bleach solution to collect their eggs. Eggs were deposited onto OP50-seeded NGM plates for 14 h to develop to L1 larvae. L1 larvae were anesthetized with levamisole on 2% agar pads for imaging with a Nikon Eclipse Ti2-E microscope using differential interference contrast microscopy. All larval lengths were measured by the same experimenter using NIS-Elements software.

**Statistical analyses:**

Differences in late-mating success across all replicate populations for each strain were determined using Cochran-Mantel-Haenszel tests with Benjamini-Hochberg corrections applied to adjust for multiple comparisons. Woolf tests were first run to test for homogeneity between replicate populations, to ensure that using the Cochran-Mantel-Haenszel test was statistically valid. Two-tailed paired t-tests were used to test the effects of fasting on average larval length for each strain. Statistical analyses were performed in GraphPad Prism 10 and R Studio with R version 4.5.0.

## Reagents

**Table:** 

**Strain**	**Genotype**	**Available from**
N2	*Caenorhabditis elegans* (wild type, var Bristol)	CGC
HRN666	*wrt-10 ( aus36 ) II *	CGC
CB4108	*fog-2 ( q71 ) V *	CGC
OP50	*Escherichia coli*	CGC
